# Non-compliance with and non-enforcement of UK loot box industry self-regulation on the Apple App Store: a longitudinal study on poor implementation

**DOI:** 10.1098/rsos.250704

**Published:** 2025-05-28

**Authors:** Leon Y. Xiao, Mie Lange Lund

**Affiliations:** ^1^ School of Creative Media, City University of Hong Kong, Hong Kong; ^2^ beClaws.org, London, UK; ^3^ Independent Researcher, Odense, Denmark

**Keywords:** loot boxes, video games, industry self-regulation, consumer protection, information disclosure, video gaming regulation, interactive entertainment law, information technology law, social corporate responsibility, gambling-like mechanics and products

## Abstract

Loot boxes in video games can be purchased with real-world money in exchange for random rewards. Stakeholders are concerned about loot boxes’ similarities to gambling and their potential harms (e.g. overspending money and developing gambling problems). The previous Conservative UK Government decided to first try relying on industry self-regulation to address the issue, rather than to impose legislation. These self-regulations have since been published by the industry trade body, Ukie (UK Interactive Entertainment). Responding to many stakeholders’ desires for a transparent and independent assessment of their implementation, we assessed companies’ compliance with three empirically testable measures and also whether the rules were actively enforced. The 100 highest-grossing iPhone games were longitudinally examined both prior to the self-regulations coming into effect on 18 July 2024 (i.e. between January and June 2024) and after to check for potential improvement (i.e. between July and December 2024). Disappointingly, widespread non-compliance and non-enforcement were observed. Among games with loot boxes, none (0.0%) sought to obtain explicit parental consent prior to enabling loot box purchasing by under-18s. Only 23.5% disclosed loot box presence, and the few disclosures were all visually obscured and difficult to access. A mere 8.6% consistently disclosed the probabilities of obtaining different rewards for all loot boxes found. The rules were not enforced, contrary to Ukie’s promise: all of the games that were non-compliant before the self-regulations came into effect remained non-compliant many months later, despite Ukie and the Apple App Store having been provided with evidence of the contraventions and put on notice to delist those games if remedial actions were not forthcoming. Because Ukie itself cannot enforce these self-regulations, platforms (e.g. app stores), the advertising regulator and the consumer protection regulators must better enforce pre-existing rules to ensure adequate consumer protection as already promised. Video games and loot boxes are no longer novel; laws that apply to all industries must also be enforced against this one. Governments are advised against relying on industry self-regulation, especially after repeated demonstrations of its many failings. Stricter legal regulation of loot boxes should be adopted. Preregistered Stage 1 protocol: https://doi.org/10.17605/OSF.IO/3KNYB (date of in-principle acceptance: 25 March 2024).

## 1. Introduction

Loot boxes are products inside video games that players can buy to obtain random rewards. Some non-paid loot boxes may be obtained without spending real-world money (e.g. through performing various in-game tasks) [1]. However, the present study focuses on paid loot boxes that players spend real-world money to purchase either directly or indirectly by spending money to purchase ‘premium’ in-game currency that can then be used to buy loot boxes. Hereinafter, references to ‘loot boxes’ refer only to ‘paid loot boxes’ unless otherwise specified. Importantly, a ‘loot box’ does not need to be visually portrayed as a box: any in-game purchase involving real-world money with any randomized elements satisfies the definition [2].

Stakeholders (e.g. consumers, parents and advocacy groups) are concerned about loot boxes’ structural and psychological similarities to gambling [3] and how vulnerable consumers (e.g. children and people experiencing problem gambling harms) might be at risk of overspending money [4–6]. Policymakers around the world are considering potential regulation [7–10], and a few countries have already taken action [11]. For example, in 2018, Belgium applied pre-existing gambling law to attempt to ‘ban’ loot boxes as unlicensed illegal gambling [12]. However, this intervention has been poorly enforced, such that 82 of the 100 highest-grossing iPhone games on the Belgian Apple App Store in mid-2022 continued to sell loot boxes in exchange for real-world money [13]. The Belgian experience demonstrates that a traditional gambling regulator that was originally resourced (e.g. in terms of public funding and manpower) to monitor only the traditional gambling industry would not be capable of also regulating the video game industry (which is composed of many smaller operators often based in foreign jurisdictions) without significantly more additional support, monetary and otherwise.

For other countries, amending gambling law to additionally include loot boxes and thereby place them within the purview of the gambling regulator is therefore likely unworkable without substantial investments towards that end. Such investments may be viewed as an unjustifiable expenditure of public money [14, paras 248–250]. Recognizing that, the UK Government (specifically, the Department for Culture, Media and Sport (DCMS)) decided in July 2022 to ask the industry to try better self-regulating loot boxes and addressing relevant concerns first, rather than to immediately impose legislation [14]. The Government did promise that it ‘will not hesitate to consider legislative options’, if video game companies and platforms do not ‘improve protections for children, young people and adults’ and if ‘tangible results’ cannot ‘begin to be seen in the near future’ [14, para. 32]. These self-regulatory rules, presented as 11 principles, have since been published 1 year later by Ukie (UK Interactive Entertainment), the national video game industry trade body, on 18 July 2023 [15], with support from the Government [16].

Besides the aforementioned Belgian example, previous research has also found that companies’ compliance with various other loot box-related regulations has been poor. In China, where companies are required by law to disclose the probabilities of obtaining various random rewards from loot boxes [17], most high-grossing games were found to have complied sub-optimally by choosing methods of displaying the disclosures that lacked visual prominence and were difficult to access [18]. Indeed, industry self-regulation of loot boxes is not a new concept and has already been attempted for several years to dubious benefit. The potential underlying efficacy of the interventions has not been scientifically proven and has never been measured since implementation (although this easily could have, and should have, been done by the industry to inform all stakeholders and improve public confidence). Importantly, many of the highest-grossing games were found to have been non-compliant, and relevant platforms and rule-makers did not appear to have actively monitored compliance, nor punished non-compliance, with previous industry self-regulation. For example, in mid-2021, 36% of the highest-grossing iPhone games containing loot boxes were found to have failed to disclose probabilities, as required by Apple App Store’s platform rules, seemingly with impunity [19].

The North American (ESRB; the Entertainment Software Rating Board) and European (PEGI; Pan-European Game Information) age rating organizations’ mandated loot box presence warning label was not properly implemented through the IARC (International Age Rating Coalition) system, such that 71% of popular games containing loot boxes did not bear the label on the Google Play Store and thereby failed to inform consumers about the potential risks [20]. Many games were also identified as unlabelled on other storefronts operated by Epic Games, Nintendo, Sony and Microsoft [21]. A number of unlabelled games have since been duly labelled, for which some credit is due to the self-regulatory age rating organizations; however, that was done only in response to external academic scrutiny and after being explicitly requested, in the absence of which, those games would likely have remained incorrectly unlabelled even today [22].

Prior research has demonstrated that loot box regulations, particularly industry self-regulatory ones, were poorly complied with in the past. Accordingly, reasonable doubt can, and ought to, be cast on whether companies will comply with the newly proposed UK loot box industry self-regulation. Many stakeholders are interested in a transparent and fair assessment of the implementation of the Ukie self-regulatory principles. Not every principle contained therein is capable of empirical study. For example, Principle 7 is to support the implementation of the *Video Games Research Framework* [23], which is a UK Government document intended to promote better research into video games and related issues. Similarly, Principle 9 is a commitment to adopt more lenient refund policies when it can be demonstrated that in-game purchases were made without parental consent or knowledge. Such principles would be welcomed by all stakeholders without controversy, but compliance with them is difficult to quantify or objectively measure against a predetermined standard.

However, three principles are empirically testable (and indeed two of them have already previously been so assessed [18–21]). First, Principle 1 demands that the purchase of loot boxes with real-world money by under-18s is to be restricted such that it may only be done with parental consent. (This is the only ‘new’ requirement that has not already been otherwise introduced; the following two requirements should already have been adopted elsewise as detailed below.) Second, Principle 4 requires companies to disclose the presence of paid loot boxes to consumers prior to purchasing or downloading the game using, *inter alia*, the relevant PEGI presence warning label [24]. Third, Principle 5 states that companies must make probability disclosures informing players of their likelihood of obtaining various random rewards from loot boxes.

Companies have been given a 12 month implementation period (starting from 18 July 2023) to adopt these measures [15]. In other words, one cannot say that a game that continues to permit loot box purchasing by under-18s without parental consent is actually non-compliant with Principle 1 until 18 July 2024. Notwithstanding, the disclosure of loot box presence to consumers prior to purchasing and downloading in any advertising of a video game (now, arguably misleadingly, presented through Principle 4 as a supposedly new measure) has already been required by advertising regulations, as clarified in the *Guidance on advertising in-game purchases* published in September 2021, which is enforced by the relevant regulator, the Advertising Standards Authority (ASA) [25]. The first author has since complained to the ASA about games that were non-compliant, and the ASA Council has held in two separate rulings that companies that do not disclose the presence of loot boxes on Apple [26] and Google [27] store pages are breaching advertising *law*. Therefore, irrespective of the implementation process, games should already be compliant with the essence of Principle 4; otherwise, they are advertising illegally. Similarly, many platforms, including the Apple App Store, have required the disclosure of loot box probabilities since 2019, if not earlier [28–30], as now also expressed through Principle 5 (again, arguably misleadingly as if this is a new proposal). Hence, non-compliance with Principles 4 and 5, even prior to the end of the 12 month implementation period or 18 July 2024, would contravene other existing regulations and be reprehensible.

The UK Government [16, para. 23] and Ukie [15] have both expressed that progress should be monitored and periodically reviewed during the implementation process. Relevant civil servants have informed the first author that it would be beneficial for independent, transparent scrutiny of the compliance with these measures (which is one reflection of their potential efficacy, as even an effective measure that is not complied with would be ineffective) to be conducted six months after the publication of these principles (i.e. around January 2024) and then again following the implementation period (i.e. around July 2024). This would complement any assessments that the industry, represented by Ukie itself, might conduct and publish.

*Research Question* 1: *Are the 100 highest-grossing iPhone games complying with three separate aspects of the UK loot box industry self-regulation?*

This was assessed by checking whether all highest-grossing iPhone games containing paid loot boxes in the 18 January 2024 sample and the 18 July 2024 sample will (i) prevent loot box purchasing by under-18s unless parental consent has been provided; (ii) disclose loot box presence; and (iii) make loot box probability disclosures.

*Research Question* 2: *Do platforms and rule makers enforce their own self-regulation and punish non-compliant companies?*

This was assessed by checking whether all highest-grossing iPhone games containing paid loot boxes in the 18 January 2024 sample that will not disclose loot box presence or make loot box probability disclosures will either have done both, or have been delisted from the UK Apple App Store, by 18 July 2024.

## 2. Method

The lists of the 100 highest-grossing games for the iPhone platform in the UK on 18 January 2024 and on 18 July 2024 were separately collated through data.ai, a leading analytics company. These two lists formed the samples, which were individually studied at two separate points in time following each aforementioned date. It was preregistered that the studying of each sample would be done ‘immediately following each aforementioned date’. However, due to resource constraints, it transpired that the research process for each sample took multiple months to complete. Importantly, all games in the 18 January 2024 sample were studied prior to 18 July 2024. If any game in the 100 highest-grossing lists would no longer have been available for download from the UK Apple App Store by the data collection period, then it would have been excluded from the sample and replaced with the next highest-grossing game (e.g. the first unavailable game would have been replaced with the 101st highest-grossing game). However, all games remained available, and so no game was replaced, although certain aspects of a few games could not be studied, as explained below. It was preregistered that ‘The results in relation to each list/sample will be separately reported in two studies in order to ensure that the results may be promptly published to assist in policy implementation when they still remain relevant’. However, this should have been amended when revising the stage 1 registered report per reviewer and recommender comments. In fact, the study results have not been separately reported but are all contained herein; however, the headline results in relation to the first 18 January 2024 sample were sent to relevant stakeholders on 25 June 2024 alongside a request for stricter enforcement, to inform the policy implementation process as detailed below.

Previous studies assessing loot box prevalence and compliance with presence warning and probability disclosure requirements have focused on the 100 highest-grossing games. That sample size and sampling method are reasonable and justified, due to resource constraints (mostly on researcher time) and given that stakeholders (e.g. parents and policymakers) are far more interested in the situation concerning popular games that many players have demonstrably spent money on, rather than that of obscure titles that may only be downloaded a handful of times per year. With that said, a key limitation must now be conceded with this sampling methodology. Previous research has repeatedly focused directly or indirectly on the 100 highest-grossing games [13,18–20]. The games on that list have not changed significantly over the years (although some entries do get replaced by newly released titles occasionally, they would still be relatively high-grossing games, e.g. be within the 500 highest-grossing games), meaning that previous research has studied certain games multiple times and, importantly, had publicly identified them as being non-compliant with pre-existing regulatory requirements that the present study is also assessing, such as not making probability disclosures or not disclosing loot box presence. Those previous research efforts are known to have directly caused the companies behind some of those games to take remedial actions to become compliant or to be forced to do so by age rating organizations [22]. Some of these same games would likely be included in the 100 highest-grossing list again on the data collection dates of the present study, meaning that, although they may now be identified as compliant, that was already guaranteed by (and indeed only due to) previous external intervention. It cannot be known whether those now-‘compliant’ games would have been so without that external interference. This means that the compliance rates among the highest-grossing games are likely to now be artificially higher than those among all other games. Alternatively, randomly sampling 100 games from the 500 highest-grossing games also would not completely remove this bias because some games that were previously studied and whose compliance was artificially affected would have fallen below the 100th rank but still remain within the top 500. Those lower-ranking and less popular games would also affect significantly fewer players and therefore be less concerning to stakeholders. Indeed, it remains valid to simply sample the 100 highest-grossing games because the findings would be the most practically informative and relevant as they show the situation as a consumer would encounter it. The artificial interventions have already happened and thereby affected the average consumer experience (hopefully positively), so although any findings would no longer be entirely ‘natural’, such findings remain the most useful. The present results simply must not be overinterpreted as indicative of the compliance rates among less popular games (which are likely to be lower) or how they would have been had there been no previous intervention.

The focus on the Apple App Store platform is predominantly due to resource constraints on the first author’s time. Ideally, the situations on other platforms (e.g. the Google Play Store and the stores of consoles like the Sony PlayStation, Microsoft Xbox and Nintendo Switch) would also be assessed. However, previous research has suggested that the loot box issue is more concerning on mobile platforms than on PC and console platforms: this is because there is significantly more content on mobile platforms (which makes compliance and enforcement more difficult), and the prevalence rate of loot boxes is also significantly higher there [20]. There are also further complications with potentially studying the Android mobile platform specifically. First, games can be installed through many different storefronts (e.g. the Samsung Galaxy Store, HUAWEI AppGallery, etc., which are not covered by the self-regulation, as it applies only to certain explicitly listed platforms). Second, games may be easily installed directly with a .apk (Android Package) file that may not be the UK-compliant version. Therefore, a study of only the Google Play Store does not fully reflect the experience of a (child) consumer using Android devices. The present study is intended to focus limited resources on providing a fair perspective on the iOS platforms, where the Apple App Store solely dominates as it is the only permitted app store for the operating system in the UK [31]. This would also provide data comparable to those of a previous 2021 study on iPhone probability disclosures in the UK [19]. Finally, the versions of the game available on the Apple App Store and Google Play Store should, in theory, be substantively identical, and the highest-grossing lists for the two platforms overlap significantly, so the present results should be broadly transferable. For example, if the iPhone version made probability disclosures, then the Google Android version probably would have done as well.

The following variables were measured.

### 2.1. Apple age rating

This was copied from the relevant age rating information displayed on the game’s UK Apple App Store page. No game was excluded due to its age rating because Apple’s highest age rating is 17+ and the Ukie principles apply to all young people under 18. Therefore, 17-year-olds can play all games available on the Apple App Store but are still supposed to be protected by the self-regulation.

### 2.2. Presence of paid loot boxes

Each game was downloaded from the UK Apple App Store and played for an hour to identify whether paid loot boxes (as defined in Annex B of the Ukie self-regulation, which aligns with the present study’s and the ESRB’s definition as set out in §1 above [2]) are being implemented and sold in exchange for real-world money or premium in-game currency that could in turn be bought with real money. If multiple loot boxes were found within that hour, then they were each separately noted. Screenshots were taken of any found loot boxes.

One hour of ‘playing’ the game meant that, from downloading and starting the software, the coder used their best endeavours for 60 min to unlock as many aspects of the game and gain access to as many in-game purchasing offers as possible: for example, the coder chose to access the in-game store where loot boxes are presumably sold as soon as able, including by skipping unnecessary story elements. Our previous research using this methodology has acknowledged that the detection rate of loot boxes is not 100% because there are likely games that only begin to sell loot boxes many hours after the player starts playing and because loot boxes might simply be missed by the researcher [19, p. 12]. This 1-hour time limit is justified by resource constraints on the coder’s time. In addition, based on previous research, this method should be sufficient to detect at least 80% of games with loot boxes (assuming that every game contains loot boxes in the UK, which is most likely untrue, so the true detection rate is higher) [13]. The percentage rate of games found to contain paid loot boxes within 1 h of examination is referred to as the ‘prevalence rate’ of loot boxes (as has been done in the past), even though more accurately, it would be the prevalence rate when only 1 h has been spent examining the game, and the true prevalence rate is therefore likely higher.

### 2.3. Presence of technical measures to prevent loot box purchasing by under-18s

When playing each game, if and whenever prompted by the game to answer any questions relating to age (such as ‘how old are you?’ and ‘in which year were you born?’), an answer that would make the user appear to be 17 years old was provided. This age was chosen because some games on the Apple App Store platform are given the highest age rating of 17+, which should render them unavailable for download by younger users. A 17-year-old can download and play them, but they also remain under 18 for the purposes of the Ukie loot box industry self-regulation, such that their loot box purchasing should be restricted until parental consent is provided. The purchase of paid loot boxes (or premium in-game currency used to purchase loot boxes) was attempted to check whether this could be done without parental consent or knowledge on a user account that purportedly belonged to a 17-year-old. A game would be deemed as having complied with Principle 1 if the aforementioned paid loot box (or premium in-game currency) purchasing attempt is unsuccessful. The additional reference to premium in-game currency presented in parentheses was not preregistered due to an oversight.

Importantly, the operating system-level spending control feature that Apple provides for parents (‘Ask to Buy’ [32]) was not accounted for by the present study. Activating this would blanketly require under-18s to send requests for approval to their parents for *all* in-game purchases (regardless of whether they are loot box purchases), app store purchases and even app store downloads of ‘free’ games. Under-18s must wait until these are approved before the transaction can take effect. That feature is undoubtedly valuable for parents and other caretakers wanting to better monitor and manage their child’s video game spending and should be used by them, but the present study is concerned with individual game-level compliance and interventions that specifically relate to loot boxes by highlighting that a purchase is potentially problematic because it is a loot box purchase. Broad, platform-wide spending control mechanisms like Apple’s Ask to Buy often fail to provide specific information about loot boxes because loot boxes are very rarely directly purchased with real-world money and often must be purchased using (premium) in-game currency (e.g. ‘Green Gems’) that is in turn bought using real-world money. Indeed, representatives of the video game industry, including Ukie [33, p. 9, para 43] (see also [34, p. 13]), have previously argued that games that directly sell loot boxes, rather than sell them through an intermediary premium currency, are confusing for the player and arguably in contravention of Principle 4 of the Office of Fair Trading’s principles for online and app-based games [11,35]. The payment request to parents would therefore merely appear as a request to purchase in-game currency with real-world money and provide no information on what that currency would then be used for (e.g. purchasing loot boxes). No further notification would be provided by the Ask to Buy system to the parent when those Green Gems are then used in-game to purchase loot boxes. This latter instance is where an in-game intervention asking for parental consent to a loot box purchase (as envisioned by the Ukie self-regulation) is expected to occur.

To further illustrate, by relying on platform-level controls only, it would require the parent to ask the child what they intend to spend the Green Gems on (when the Ask to Buy request for that transaction appears), before the real-money-to-Green-Gems transaction takes place, for them to find out that loot boxes would be purchased. The child might be undecided as to how they want to spend the Green Gems, and the child might also not understand that, with those Green Gems, they would be buying a gambling-like ‘loot box’ that is seen as problematic (as many of these products are not advertised as such) or be untruthful as to how they intend to spend the Green Gems. The game company must directly communicate the fact that loot box purchasing by a child is taking place to the parent. Alternatively, doing this through the child as an intermediary is not a dependable or acceptable proposal. In short, the platform-level controls (assuming that they are turned on) effectively restrict the first premium currency transaction using real-world money but never the second loot box purchasing transaction using in-game premium currency (which is where the intervention should take place). Platform-level controls may be deemed sufficient for games where the payment request is for loot box-like mechanics directly and that request clearly explains how the mechanic works and any associated concerns. However, given that nearly all games do not offer this, relying solely on this measure would be unwise. For payment requests to purchase premium currency, Apple may consider allowing (but has not yet allowed) games to append information on how that premium currency might then be spent on loot boxes and such mechanics and outline the potential related concerns. However, until that is uniformly and satisfactorily done, a parent cannot trust the Ask to Buy feature alone to protect their child.

Another point is that platform-wide parental control would also require the parent to activate it. These may be turned on by default in some instances, but regulation must not proceed on the unreasonable assumption that all parents already have this turned on for every child. It would not be right to place that burden on parents. A child may also engage with a game downloaded on a parent’s or the family’s device, in which case platform-level controls are not active. Multiple layers of protection should be provided. Indeed, had robust parental controls already been widely utilized, then no further regulation (including the Ukie self-regulation) would be required. It is precisely because of the potential failings of pre-existing parental control features that the Ukie self-regulation is being newly introduced to directly address the loot box issue. It is therefore reasonable to expect individual games to be taking new actions and making interventions inside the game. The drafters of the self-regulation surely must not have deemed pre-existing platform-wide parental controls to already be sufficient, as those have already been available for many years, and so the self-regulation would then be proposing nothing new.

### 2.4. Presence of presence disclosures

For each game found to contain paid loot boxes, its Apple App Store product page was reviewed to attempt to find a disclosure of loot box presence, such as the PEGI warning label of ‘In-game Purchases (Includes Random Items)’ [20,24] or some text describing the availability of paid loot boxes. Any disclosure, however difficult to find and access and however phrased, was recognized as a disclosure having been made as long as it could reasonably have been so interpreted because the self-regulation merely requires that this be done and not that it be done visually prominently or informatively [15, p. 5]. Nonetheless, it was preregistered that different methods of disclosure would be categorized; however, in fact, only one category was found. A game is deemed to have complied with Principle 4 if a loot box presence disclosure could be found.

### 2.5. Presence of probability disclosures

In relation to each type of loot box found in each game, a corresponding probability disclosure was searched for in-game. No external searches were conducted (e.g. through a search engine) for disclosures that may have been available only on websites and were not linked from within the game because the relevant Ukie guidance makes clear that disclosures should be ‘easily [accessible]’ [15, p. 5] and any website-based disclosures (although permitted) should also be sign-posted from within the game itself [15, p. 15]. All found probability disclosures were screenshotted, and the process for accessing them from the loot box purchase screen was documented. Any disclosure format, regardless of its visual prominence or ease of access, was recognized as a disclosure having been made, because even though Principle 5 encourages ‘easily [accessible]’ and ‘clear and simple’ probability disclosures, those qualities are subjective to a certain degree. Different methods of disclosure were categorized. A game is deemed to have complied with Principle 5 only if a corresponding probability disclosure could be found for every identified loot box type.

### 2.6. Changes in compliance following initial study and reporting to Apple and other stakeholders

Any non-compliance with Principles 4 and 5 found among the 18 January 2024 sample was reported to Apple and other stakeholders (e.g. DCMS and Ukie) for enforcement actions to be taken (e.g. an ultimatum to comply by a certain date, failing which the game would be removed from the UK Apple App Store for contravening platform rules or advertising regulations). Any non-compliance with Principle 1 among the 18 January 2024 sample was also reported, but no further action was requested given that a game is required only to comply with that measure by 18 July 2024. For games that were included in the 18 January 2024 sample and were found to have been non-compliant with any one of the two principles, they were re-examined alongside the 18 July 2024 sample (if they were not already included in that sample), to check any potential changes in compliance (e.g. (a) having since complied or (b) having since been delisted). (The preregistration mistakenly stated that games that were non-compliant with Principle 1 among the 18 January 2024 sample would also be reviewed; however, this was a typo and was not done because this would not reflect upon whether enforcement actions were being actively taken as this rule was not in force at the relevant time and so could not be ‘enforced’. Indeed, no enforcement in relation to that measure was requested when the January results were sent to relevant stakeholders on 25 June 2024.)

### 2.7. Date and time of data collection

The date and time, based on UK time, on and at which the game was examined were recorded.

The ‘compliance rate’ with each loot box self-regulatory measure is calculated as follows:


$$\frac{Games\;containing\;loot\;boxes\;and\;complying\;with\;the\;relevant\;measure}{Games\;containing\;loot\;boxes}.$$

Even though some games might be inaccurately marked as not containing loot boxes even though they do using the present methodology of examining the game for 1 h only (because the loot boxes would only become available for purchase after more than 1 h of gameplay), the compliance rates with various regulatory measures would not be affected because games assumed to not contain loot boxes would have been excluded. The relevant compliance rates reflect the true situation among the games containing loot boxes that were actually tested.

For each measure assessed for Research Question 1, a compliance rate of at least 95% would have been interpreted in §4 below as near-perfect and satisfactory compliance. This 5% leeway (from a perfect compliance of 100%) was permitted as a type 1 error control measure to account for potential false positives. A compliance rate that is ≥80% but <95% would have been interpreted as a measure having been mostly complied with but needs some improvements. A compliance rate that is <80% would have been interpreted as the measure not having been adequately complied with and needing significant improvements to achieve the regulatory aim. In addition, had the compliance rate with a specific measure improved from one band into the next (e.g. from <80% to ≥80%) when the 18 July 2024 sample was compared with the 18 January 2024 sample, then the authors would have commented positively on how compliance has improved. These cut-offs were used previously and are based on the first author’s intuition as to what consumers, policymakers and independent researchers would likely deem acceptable or not [20,21]. These cut-offs were preregistered to ensure that the first author’s subsequent interpretation would not be affected by the compliance rates that were eventually found. This is because a certain compliance rate is open to multiple interpretations by various stakeholders and indeed by the same person. For example, one might subjectively interpret a 60% compliance rate as either poor or satisfactory: an industry representative might say it is good, while an advocacy group in favour of banning loot boxes might view it as terrible; both sides are arguable. However, any flexibility in potential interpretation by the first author was hereby eliminated through preregistration of the aforementioned cut-offs. (As it transpired, all the compliance rates found fell far below 80.0%, and so most of these cut-off rules did not become relevant.)

For that same reason, the first author invited stakeholders (specifically, DCMS and Ukie) to preregister how they would interpret different potential results that may be found in the present study. However, both have refused. DCMS stated on 1 December 2023 in response to the first author’s request that it is ‘extremely cognisant of the need for a high rate of compliance and suitable tracking of it, but [DCMS has] made a recent public statement on loot boxes [referring to its 18 July 2023 statement supporting and approving the Ukie self-regulations upon their publication, which did not set out what degree of compliance would be deemed satisfactory by the UK Government [16]] and are not planning to say anything further publicly at this point while [it continues] to work behind the scenes with academics and industry’. The first author also understood that there may also have been some hesitancy on the part of civil servants in purporting to bind not just the then current government’s interpretation but also the next government’s (which might well be formed by and, as it transpired, was indeed formed by the opposing political party, which may have different views on what degree of compliance is acceptable).

Ukie’s refusal of the first author’s request on 18 December 2023 stated, first, that it did not think the assessment should occur before the end of the 12 month implementation period and, second, that the first author’s proposed method of testing Principle 1, which specifically excludes platform-level controls, was unacceptable. The first author was willing to accept Ukie’s refusal in relation to Principle 1 (prevent under-18s from purchasing loot boxes without parental consent). However, the reasons provided by Ukie did not apply to the testing of Principles 4 (presence disclosures) and 5 (probability disclosures), which are both already required by other regulations, irrespective of the implementation period of the Ukie self-regulatory principles, and whose assessment methods are objective and have not been objected to. The first author therefore replied and asked Ukie to then instead preregister its potential interpretations for Principles 4 and 5 only. Ukie did not respond. Unfortunately, these negative responses mean that there is a lack of transparency and accountability to the public. It is also unfortunate for the industry that it does not have a clear target to meet and may still be regulated against, despite many members having used their best endeavours.

For Research Question 2, we would have concluded that the self-regulations are being properly enforced if *all* games among the 18 January 2024 sample that were non-compliant with either Principle 4 or 5 would either have complied with both measures or have been delisted from the UK Apple App Store by 18 July 2024. Otherwise, we would make the opposite conclusion and criticize relevant stakeholders for not strictly enforcing platform rules, advertising regulations and the Ukie self-regulatory principles. The only exception would be if a game has since stopped selling loot boxes, in which case that game was excluded when answering Research Question 2. The expectation that 100% (rather than 95%) of games would either become compliant or be delisted is justified on the basis that a list containing all relevant non-compliant games was provided to the stakeholders to take enforcement actions. Any potential Type 1 error would have been eliminated by how the Apple App Store and/or the relevant video game companies was given the opportunity to provide evidence that the game does not contain loot boxes or have already made the relevant disclosures, so a further 5% of leeway (given above for other assessments) is not appropriate here.

To further address the issue of how the compliance rates among the highest-grossing games may have been affected by previous external intervention, the compliance rates for each loot box self-regulatory measure are also separately reported for games that have previously been studied and those that have not been.

In accordance with the *Danish Code of Conduct for Research Integrity* [36], as adopted by the IT University of Copenhagen, the present registered report did not require research ethics assessment and approval because no human participants or personal data were involved and only publicly available information was examined and recorded.

## 3. Results

The results for the 18 January and 18 July 2024 samples (hereinafter, the ‘January sample’ and ‘July sample’, respectively) are reported individually, and longitudinal insights are then discussed.

### 3.1. Prevalence of loot boxes and Apple age ratings

Among the 100 highest-grossing games on the UK Apple App Store in January 2024, 83.0% contained loot boxes, while 81.0% of the highest-grossing games did in July 2024. Breakdowns of the game titles by age rating are shown in table 1. There were no meaningful differences between the two samples.

**Table 1.** Apple App Store age rating of games containing loot boxes in the January and July samples (cumulative).

age rating	total games	games with loot boxes	prevalence of loot boxes
**18 January 2024 sample (*****n*** **= 100)**			
4+	31	22	71.0%
9+	45	34	75.6%
12+	85	69	81.2%
17+	100	83	83.0%
**18 July 2024 sample (*****n*** **= 100)**			
4+	34	22	64.7%
9+	49	36	73.5%
12+	84	67	79.8%
17+	100	81	81.0%

### 3.2. Preventing under-18s from purchasing loot boxes?

#### 3.2.1. January sample

To implement any protective measures in relation to under-18s specifically, the game must find out whether the individual player in question is under 18 years of age. In relation to the January sample, only 35 of 100 games (35.0%) conducted any manner of age verification. There were broadly two categories of age verification: (i) asking players to affirm that they are over a certain age (used by 9 of 35 games that verified the player’s age (25.7%)) and (ii) asking players to input an age or birthdate (used by 26 of 35 games that conducted age verification (74.3%)). Both categories are based on so-called ‘self-declarations’. The company accepts whatever information the player provides as true and proceeds on that basis. For context, it should be noted that, in the UK, this type of age verification, which can be easily circumvented, was explicitly deemed as insufficient and not constituting a satisfactory age verification method under Section 230(4) of the Online Safety Act 2023 (which likely applies to many online video games that allow for interactions between users per Section 3(1) of that Act), for example. Taking that into account, no video game (0.0%) conducted effective age verification that is not based on self-declarations before permitting gameplay and in-game purchasing, e.g. government-issued photo ID and biometric verification through a third-party service provider. *Roblox* was the only game that provided an option for the player to verify their age using their ID to unlock certain features if desired [37], but loot box purchasing was a feature that was always accessible irrespective of whether the ID verification process was completed. The *Presence of technical measures to prevent loot box purchasing by under-18s* variable could not be coded in relation to *EA SPORTS FC 24 Companion* because the player must play the console and PC game, *EA SPORTS FC 24*, which the *Companion* game supports for a significant amount of time (above and beyond the 1 h of coding time allocated) before the ability to make in-game purchases in the *Companion* game would become available and thereby assessable.

Assuming that the player did not lie about their age and honestly stated their age to be 17 or provided a birthdate that meant that they would have been recognized as 17, it was then not possible to play a small minority of games (10 of all 100 games (10.0%); and 8 of 83 games with loot boxes (9.6%)). These games either asked the player to affirm that they are over 18 (as in *Coin Master*) or would have blocked the player from accessing gameplay if they provided an age verification answer that meant they were deemed as being under 18. However, it was generally easy to circumvent these measures. For *Coin Master*, the player just had to tap on the ‘CONFIRM’ button (which was in fact the only available action the player could take, other than closing the software), irrespective of whether they were actually over 18. Many players may even have entirely missed the relevant age requirement message due to an established habit of accepting all terms and conditions shown upon first starting a game. Even when gameplay blocking measures were implemented for ‘wrong’ age verification answers, the games often allowed players to immediately input a different answer if they previously inputted an ‘incorrect’ answer for the age verification question (as in *Toy Blast*); after a countdown (e.g. of 2 h, as in *Call of Duty: Mobile*); or immediately after the game is deleted and then reinstalled (as in *June’s Journey: Hidden Objects*). Only in two games (*Bingo Blitz* and *Solitaire Grand Harvest*) was the player not able to reattempt the age verification process through an easy circumvention method. Neither restarting the game nor uninstalling and reinstalling the game allowed the player to circumvent the gameplay block. This meant that the two games collected and stored some information about the player or their device either on the company’s servers or locally on the player’s phone. This may have been in breach of data protection law as the player did not consent to their personal information (age and device identifier) being collected, processed and retained for this purpose, although the company may instead rely on how, despite lacking consent, it has a legitimate purpose in collecting, processing and retaining such data (to conduct effective age verification and enforce age limits in order to protect children). Only a ‘factory restore’ of the phone or playing on a different device entirely was sufficient in forcing these two games to allow the player to reattempt the age verification process again and potentially lie to circumvent it.

The preregistered method did not contemplate how some games would be rated suitable for 17-year-olds or even younger children (e.g. those aged above 4) per the Apple App Store but then block them from actually playing the game through an age verification process after the game is downloaded as set out above. This was because companies acting in this manner likely breached consumer protection law. For example, *Toy Blast* was rated suitable for children aged 4+ on the Apple App Store and was advertised as such by displaying this age rating on its product listing and using cartoon-like aesthetics, but the game would not actually provide service unless the player certifies that they are over 18. This means that the age rating information provided on the product listing was misleading and may cause consumers to make a transactional decision that they otherwise would not have (i.e. download a video game that they could not actually play, which they would not have downloaded if they were duly made aware of its true age requirement).

Accordingly, it is open to interpretation whether the games that conducted self-declaration-based age verification (which would not be viewed as effective age assurance in the UK under the Online Safety Act, for example) and blocked 17-year-olds from accessing the game complied with Principle 1 of the Ukie self-regulation or not. A strict interpretation of the preregistered method would require that all games that blocked gameplay for 17-year-olds be excluded from the analysis, as it was simply not possible to assess whether these games would seek parental consent for loot box purchasing by an account purporting to be owned by a 17-year-old player because such an account cannot exist in the first place. Alternatively, the games that implemented a relatively robust age verification system and strictly enforced it (e.g. *Solitaire Grand Harvest*) may be viewed as compliant because an honest 17-year-old who did not lie about their age could not access the game at all, let alone purchase loot boxes within it. However, it is unclear whether the games whose age verification process was less robust and could be easily circumvented by a 17-year-old who wished to access the game (e.g. tapping on the only available button, as in *Coin Master*), which is a possibility that companies should be obliged to address and prevent, should be viewed as compliant or not.

In addition, it is unlikely that an initial, one-time-only age screening is what was envisioned by Principle 1 of the Ukie self-regulations requiring companies to ‘Make available technological controls to effectively restrict anyone under the age of 18 from acquiring a Paid Loot Box, without the consent or knowledge of a parent, carer or guardian. Technological controls shall be easy to use, activate and access and are introduced to all parents, carers and guardians through [start-up] processes and unboxing’ [15]. None of the age verification processes made an explicit reference to loot boxes and sought to obtain parental consent for their purchase.

In the interest of providing a full spectrum of potential interpretations, the complete range of potential results is disclosed. On one extreme end, as the authors believe to be the most appropriate, strictly applying the preregistered method and excluding the eight games with loot boxes that did not allow accounts belonging to 17-year-olds to participate in gameplay and *EA SPORTS FC 24 Companion* from the analysis, no game with loot boxes (0 of 74; 0%) sought parental consent before permitting loot box purchasing. The loot boxes or the relevant premium currency used to buy loot boxes were always automatically purchasable as an in-game purchase in all relevant games examined. This meant that the coder was able to access the Apple App Store payment page for the in-game purchase and could have approved the transfer of money to Apple in exchange for the contents of the in-game purchase if desired on that page. This was deemed sufficient in proving that loot box purchasing by under-18s without explicit parental consent has not been blocked through technical measures and was therefore possible. A similar approach was taken for previous research in Belgium, where the last possible step was not taken due to potential illegality [13, pp. 7–8]. When studying the January sample, the coder did not proceed with the payment. No money was transferred to Apple, which would have transferred a portion of that money to the relevant game company, except in relation to the coding of *Minecraft* (for which it was required to spend money in order to actually observe the operation of the relevant loot boxes in the user-generated content in sufficient detail as to understand it).

Alternatively, on the other extreme end, if all games that an honest 17-year-old would have been prevented from playing (irrespective of how easy it was to circumvent the relevant age verification measure) are viewed as compliant with Principle 1 of the Ukie self-regulation, then (again excluding *EA SPORTS FC 24 Companion*) 8 of 82 games (9.8%) were compliant by having prevented under-18s from accessing the game at all and thus, by implication, having also prevented them from purchasing loot boxes without parental consent. The reader is also welcome to decide that only some of the age verification processes are sufficiently robust, but that others are not because they could be too easily circumvented, which results in another possible compliance rate between the range of 0.0% to 9.8%.

#### 3.2.2. July sample

In the July sample, 39 of 100 games (39.0%) conducted age verification, all of which used self-declaration-based methods like before. Similar to the situation among the January sample, 11 of 39 games (28.2%) asked the player to affirm they are old enough, and 28 of 39 games (71.8%) asked for an age or a birthdate to be inputted. None of the games (0.0%) used a sufficiently robust age verification method that was not based on self-declarations. As before, even though a small minority of games (12 of all 100 games (12.0%); and 10 of 81 games with loot boxes (12.3%)) could not be played unless the player affirmed that they were over 18 or blocked gameplay if the player failed the age verification process by providing an answer that meant that they were too young, these measures could be easily circumvented by either simply affirming or because the player could quickly obtain another chance at providing a ‘correct’ answer by lying. Both *Bingo Blitz* and *Solitaire Grand Harvest* appeared again in the July sample, and *Bingo Blitz*’s age verification enforcement, although relatively robust, was circumvented when the game was coded again. That same circumvention method likely would have also worked previously had it been attempted then. *Solitaire Grand Harvest*’s block on gameplay after failing its age verification could not be circumvented, except through a factory restore of the whole device.

Like before, strictly applying the preregistered method and excluding the ten games that did not allow accounts belonging to 17-year-olds to participate in gameplay and *EA SPORTS FC 24 Companion* from the analysis, no game (0.0%) sought parental consent before permitting loot box purchasing. None of the games with loot boxes complied with Principle 1 of the Ukie self-regulation (requiring that companies ‘effectively restrict anyone under the age of 18 from acquiring [paid loot boxes], without the consent or knowledge of a parent, carer or guardian’), despite the rule having officially taken effect.

Alternatively, the highest possible compliance rate of 12.5% (10 of 80 games after excluding *EA SPORTS FC 24 Companion*) can be generated if all games that an honest 17-year-old would not be allowed to play are deemed as compliant. Again, the reader is invited to make their own assessment as to compliance, which could range between 0.0% and 12.5%. Unfortunately, even the highest possible compliance rate of 12.5% sits significantly far below a satisfactory compliance rate of at least 80.0% as preregistered. Companies have not implemented any measures to directly seek to obtain explicit parental consent for loot box purchasing by under-18s either before or after the Ukie self-regulation came into effect.

#### 3.2.3. July sample re-examined

After coding the July sample using the same methodology adopted for the January sample, i.e. attempting to access the Apple App Store payment page for the relevant in-game purchase offer but not actually proceeding with payment, it was realized that, hypothetically, certain interventions that a video game might implement could not have been detected using that method. Specifically, if a video game does not intervene when premium in-game currency that is used to purchase loot boxes is being purchased because that currency could also be used to purchase other things in the game that are not loot boxes, but if that game then does intervene when the player attempts to use the premium in-game currency paid for using real-world money to purchase loot boxes, this intervention could not have been encountered, unless the Apple App Store payment is confirmed and money is actually transferred to the video game company in exchange for paid-for premium currency.

This arguably reflects a lack of specificity in the original preregistered method of this registered report, which should have explicitly stated whether any payment of money would be made to Apple and the game company and considered relevant ethical issues, e.g. the appropriateness of using public research funds to purchase products that are potentially harmful to public health, even though the amount is negligible. To resolve this, all games with loot boxes in the July sample (with some exceptions as detailed below) were re-examined as to the *Presence of technical measures to prevent loot box purchasing by under-18s* variable, and a video was recorded of the whole process during which the coder purchased either loot boxes or premium in-game currency by confirming the Apple App Store payment and transferring money to the video game company and then purchasing loot boxes with the acquired premium in-game currency if applicable. No effective loot box purchase intervention that sought to seek explicit parental consent was discovered in any of the games (0.0%). *Roblox* did provide a warning and asked for the account holder’s parent or guardian to consent to the premium currency purchase before it could occur when a purchase was attempted for the first time. No further warning was given for subsequent attempts. However, that is a self-declaration that could be easily circumvented, and no explicit and specific consent was sought for any loot box purchasing that could then be done using the acquired premium currency.

For this re-examination, *EA SPORTS FC 24 Companion* could not be coded for the same reason stated above; *Matchington Mansion* could not be coded because the previously identified loot box features were apparently removed since the initial coding for the July sample and could no longer be found. *The Simpsons: Tapped Out* could not be coded because the game ceased operation after the initial coding for the July sample [38]; and a purchase was not made in *Minecraft* again as this was already done through the original coding process as described above due to the game’s unique circumstances. This subsequent review process also uncovered how age verification was added to *MONOPOLY GO!* and loot box probability disclosures were added to *Solitaire Grand Harvest* since their initial coding for the July sample, thus demonstrating that their coding would have been different if those games were examined at a different time. The initial July sample coding results were not amended.

### 3.3. Disclosing the presence of loot boxes in store listings

For the January sample, 8 of 83 games with loot boxes (9.6%) disclosed loot box presence on the Apple App Store product listing page for the game. For the July sample, 19 of 81 games with loot boxes (23.5%) disclosed their presence. This is a notable increase in compliance between the two data collection periods. However, the 23.5% compliance rate with Principle 4 of the Ukie self-regulation (requiring companies to disclose the presence of paid loot boxes to consumers prior to purchasing or downloading the game) remains far below 80.0% and is unsatisfactory.

There was only one category of disclosure. All games that disclosed loot box presence in both samples did so using text that formed part of the game’s description. Importantly, in all cases, it was required that a hyperlinked ‘more’ button be tapped (on the mobile version of the Apple App Store) or clicked (on the desktop PC version of the Apple App Store) before the relevant loot box presence disclosure is provided, even though, in theory, had this information been provided at the top of the game’s description, it would have been automatically shown without requiring further input from the player. The information was also generally hidden near the bottom or in the middle of a body of text and was therefore not easy to identify. The player was always required to actively do something before the information became available, which arguably meant that the information was never provided reasonably prominently, as it was not provided by default.

### 3.4. Making loot box probability disclosures

Among the January sample, 49 of 83 games with loot boxes (59.0%) disclosed probabilities for at least one loot box contained within the game. This datapoint is comparable to the previously reported mid-2021 UK disclosure rate of 64.0% [19], although the methods are not identical (there were more opportunities for games to be compliant at least once in the current study as it usually reviewed multiple loot boxes instead of only one per game, as was done before), so the current rate would likely have been slightly lower had the previous method been used. Only 11 of 83 games with loot boxes (13.3%) disclosed probabilities for *all* loot boxes found within 1 h of gameplay.

Among the July sample, 51 of 81 games with loot boxes (63.0%) disclosed probabilities for at least one loot box, but only 7 of 81 games with loot boxes (8.6%) disclosed for all loot boxes found within 1 h of gameplay. Principle 5 of the Ukie industry self-regulation requires companies to make probability disclosures informing players of their likelihood of obtaining various random rewards from *all* loot boxes contained in the game. The compliance rate of 8.6% falls markedly below a satisfactory compliance rate of 80.0%.

#### 3.4.1. Loot box probability disclosure methods

As to the methods by which the disclosures could be accessed, these are detailed in table 2 for both the January and July samples based on the loot box found in each game that disclosed using the most prominent and accessible method. Many games had multiple different loot boxes that disclosed in different ways. Other loot boxes in the same game may have disclosed using a method that was less prominent than the one listed. Previous research has defined ‘reasonably prominent’ probability disclosures as ones that were either automatically shown or were accessed by tapping on a button that explicitly referenced ‘probabilities’ or a conceptually similar term (such as ‘rates’), which made it obvious that said button would have led to the probability disclosure [18,19]. Applying this rule, only 10 of 83 games with loot boxes (12.0%) disclosed probabilities using reasonably prominent methods for at least one loot box in the January sample, and just 10 of 81 (12.3%) did in the July sample. Again, other loot boxes in these games may have disclosed using methods that were not reasonably prominent but that was not considered. For comparison, 6.7% (5 of 75) of games with loot boxes made reasonably prominent in-game disclosures in 2021 [19].

**Table 2.** Categories of observed in-game disclosures for games containing loot boxes that disclosed probabilities for at least one loot box found in the January and July samples.

number of games		summary of disclosure format
January sample (*n* = 49)	July sample (*n* = 51)	
30 (61.2%)	28 (54.9%)	immediately after tapping a small generic symbol, such as a question mark button or a ‘view details’ button, that did NOT explicitly reference probabilities
7 (14.3%)	11 (21.6%)	after tapping a small generic symbol as described above and then following at least one additional step, such as tapping another button
5 (10.2%)	2 (3.9%)	automatically displayed on the loot box purchase page without requiring any additional input from the player
5 (10.2%)	7 (13.7%)	immediately after tapping a small button explicitly referencing ‘probabilities’ or a conceptually similar term, such as a ‘detailed odds’ or a red ‘%’ button
0 (0.0%)	1 (2.0%)	after tapping a small button explicitly referencing ‘probabilities’ as described above and then following at least one additional step, such as tapping another button
1 (2.0%)	2 (3.9%)	interacting with a graphic symbol that conceptually referenced ‘probabilities’ and ‘chance’, such as a dice button
1 (2.0%)	0 (0.0%)	interacting with certain buttons NOT on the loot box purchase page

### 3.5. Were enforcement actions taken against non-compliant games?

As preregistered, the results from the January sample as to which games were non-compliant with the measures set out in Principles 4 and 5 of the Ukie self-regulations (which, as a reminder, applied even before the effective date of the Ukie self-regulations by virtue of consumer law and platform rules) were communicated to Ukie and the DCMS of the UK Government on 25 June 2024. Lists of non-compliant games were provided alongside supporting evidence (e.g. screenshots and coder notes detailing the alleged non-compliance) [39], and enforcement actions against the non-compliant games were requested [40]. DCMS confirmed receipt on the same day [41], while no response was received from Ukie. A further email was sent to both Ukie and DCMS on 4 July 2024 explaining that *8 Ball Pool* was omitted by mistake from the list but should also be included as it was non-compliant [40]. DCMS confirmed in a meeting with the first author on 31 July 2024 that the relevant lists were also received by Ukie, which DCMS understood has acted upon the information provided, including contacting the app stores and relevant stakeholders.

In total, 83 games contained loot boxes in the January sample and were obliged to comply with (i) the requirement to disclose loot box presence on the Apple App Store product listing page and (ii) the requirement to disclose probabilities for all loot boxes within the game. Only eight games (9.6%) disclosed loot box presence, meaning 75 (90.4%) did not. Only 11 games (13.3%) always disclosed probabilities, while 72 games did not (86.7%). Only two games (2.4%) were compliant with both measures prior to 18 July 2024 when the Ukie self-regulations took effect. These two games were *EA SPORTS FC 24 Companion* (which is a mobile application that could be used to purchase loot boxes for the console game, *EA SPORTS FC 24*) by Electronic Arts and *F1 Clash - Car Racing Manager* by Hutch Games. Importantly, both companies are members of the technical working group of video game companies and other related entities convened by the DCMS to design the Ukie self-regulations and should therefore be held to a higher standard [16]. For context, both companies were previously censured by the ASA, the UK advertising regulator, for illegally failing to disclose loot box presence [26,27,42,43]. In particular, *F1 Clash*’s ‘compliance’ was forced by an ASA ruling upholding a complaint made by the first author against it [26]. Importantly, these two games remained the only games that were fully compliant after 18 July 2024.

All 81 games that were previously non-compliant in at least one way remained available for download from the Apple App Store after 18 July 2024. Notably, 41 of the 50 highest-grossing games on 18 January 2024 contained loot boxes, and 40 of them (97.6%) remained in the list of the 100 highest-grossing games on 18 July 2024, thus reflecting that the mobile game market is well entrenched and dominated by older titles and that newly released games struggle to compete. Indeed, 66 of the 83 games with loot boxes that were on the 100 highest-grossing lists on 18 January 2024 (79.5%) remained on the list on 18 July 2024. Therefore, there was evidently more fluctuation among slightly lower-ranked games, but the market was nonetheless relatively well entrenched and dominated by the same games and companies. This is strong support for the contention that any newly adopted loot box regulations should apply retroactively against previously released games that remain in continued operation to support fair competition [20].

Loot boxes could no longer be found in three games in the January sample that were previously non-compliant with at least one of the two measures. The measures were therefore no longer applicable to these games, meaning that they should be excluded from the analysis. It cannot be known whether all loot boxes have truly been removed from these three games (e.g. as a compliance action taken against the UK version of these games) or whether the coder simply did not have an opportunity to observe them when the games were examined again (meaning that the loot boxes could still have been in the game, and the game, in fact, remained non-compliant).

As to the requirement to disclose loot box presence, 11 of 72 games that were previously non-compliant and continued to contain loot boxes when assessed as part of the July sample (15.3%) became compliant when their Apple App Store product listing page was checked again after 18 July 2024. At least some of these remedial actions must be attributed to other enforcement actions elsewhere in the world beyond the UK that were instigated by the first author (e.g. in Ireland and The Netherlands), as detailed in §4. Given that only a very small minority of games subsequently complied, it does not appear that consistent and strict enforcement actions, if any at all, were undertaken by Ukie and other relevant stakeholders (e.g. the Apple App Store).

As to the probability disclosure requirement, none of the 70 games that continued to offer loot boxes and were previously non-compliant (0.0%) became compliant. More concerningly, five games that were previously deemed compliant as part of the January sample were found to have contained loot boxes that did *not* disclose probabilities when they were examined again after 18 July 2024. These games either became non-compliant or, more likely with at least one game, were inaccurately deemed compliant previously due to the research method’s limitations (e.g. insufficient resources to ensure an exhaustive review of all aspects of the game).

The preregistered benchmark was that all 100% of games that were previously non-compliant should have either become compliant after 18 July 2024 or been delisted from the Apple App Store. Instead, only 15.3% of those games became compliant with Principle 4, and none became compliant with Principle 5. When combined, this means all of the non-compliant games found in the January sample remained non-compliant when reassessed after 18 July 2024, despite the first author having provided directly actionable information and logistical support (including evidence of non-compliance) to both Ukie and the DCMS.

### 3.6. Answering the research questions

To sum up, as to Research Question 1, after 18 July 2024, the compliance rates were 12.5% (at most, and arguably 0.0%) with Principle 1 (restricting loot box purchasing until explicit parental consent is given); 23.5% with Principle 4 (disclosing loot box presence); and 8.6% with Principle 5 (disclosing loot box probabilities). None of the measures were even close to having been satisfactorily complied with (i.e. a compliance rate equal to or above 80.0%), even though, for example, compliance with Principle 4 required merely a simple editing of the description for the game that appears on the Apple App Store product listing page (which should take but a few minutes to do). The vast majority of companies are not complying with consumer law, advertising regulations, platform rules and the Ukie self-regulations.

As to Research Question 2, only 14.7% of the non-compliant games that previously did not disclose loot box presence and 0.0% (i.e. none) of the non-compliant games that previously did not disclose loot box probabilities became compliant with the relevant measure after 18 July 2024. All of the games remained non-compliant with at least one rule; none became fully compliant, contrary to the expectation that all non-compliant games would have either become fully compliant or been delisted from the Apple App Store for breaking rules. Ukie, the Apple App Store and DCMS are not enforcing or not ensuring the strict enforcement of industry self-regulatory rules (i.e. the Ukie self-regulatory principles and Apple’s platform rules) on loot boxes and not punishing non-compliant companies with delistings, despite promises to do so [44]. Further, given that these two rules also apply as a matter of consumer law and advertising regulations, irrespective of the Ukie self-regulations, the relevant UK regulators (the Competition and Markets Authority (CMA) and Trading Standards, which enforce consumer law, and the ASA, which enforces advertising regulations) must also be criticized for not enforcing the law. DCMS has also failed to ensure that these other regulators with more enforcement powers do their duties.

### 3.7. Compliance rates of previously studied games

Among the entire January sample, the compliance rate with the requirement to disclose loot box presence was 9.6% for the 83 games containing loot boxes. Nearly two-thirds (66.3%) of those games (55 of 83) were previously identified as containing loot boxes in an academic study [13,18,19,45,46]. Compliance was highly unsatisfactory among both previously studied games (10.9%; 6 of 55) and games that were not previously studied (7.1%; 2 of 28). Among the 81 games with loot boxes in the July 2024 sample, 23.5% disclosed loot box presence. Over four-fifths (84.0%) of those games (68 of 81) were identified as containing loot boxes by at least one prior study (including the study on the January sample reported herein). Compliance was 27.9% among 19 of 68 previously studied games and 0.0% among 13 games that were researched for the first time.

As to the requirement of making probability disclosures for all loot boxes found within the game, compliance was 13.3% among the 83 games with loot boxes in the January sample. Seven of 55 games that were previously studied (12.7%) complied, and 4 of 28 games that were not previously studied (14.3%) also complied. The overall compliance rate among the July sample was 8.6%. Six of 68 previously studied games (8.8%) complied, while one of 13 games that were researched for the first time complied (7.7%).

Some limitations of this aspect must be disclosed. A few games (like *Episode - Choose Your Story*) were included in the samples of a previous study but were not found to have contained loot boxes back then. These were deemed as not having previously been ‘studied’ because these games’ companies were not directly put on notice by the relevant previous research to comply. The readership of prior studies and media reports further publicizing the results could not be comprehensively assessed, so it is unclear whether the fact of having been identified as having been non-compliant in a prior study would have reached the relevant company and, even if so, whether the company acted on that information by complying. A mix of various factors that are further discussed below may have or is known to have influenced compliance behaviour.

## 4. Discussion

Compliance with all measures examined is very low and highly unsatisfactory. None of the highest-grossing games sought explicit parental consent before allowing children to purchase loot boxes, and the vast majority did not disclose the presence of loot boxes on their Apple App Store listing page or disclose the probabilities of obtaining different potential rewards for all loot boxes found within the game. Relevant stakeholders (e.g. Ukie and the Apple App Store) also did not take enforcement actions to ensure that highly popular and profitable games breaking the rules would be either corrected or delisted. Companies that broke the rules were permitted to continue doing so many months later, despite their rule-breaking having been specifically highlighted to relevant stakeholders supposedly responsible for enforcement. This echoes previous research generally finding poor and unsatisfactory compliance and broad non-enforcement with loot box regulation around the world [13,18,46–50], particularly with regard to less enforceable industry self-regulation [19–21,51]. For example, in the UK, many iPhone games did not disclose loot box probabilities as required by the Apple App Store in 2021 [19], and more than 90% of social media ads for games with loot boxes did not disclose loot box presence as required [49,50]. This situation is perhaps unsurprising considering that there has been very little enforcement of the Ukie loot box rules, despite multiple regulators technically being empowered to enforce them through different means.

First, most directly, Ukie threatened that non-compliance with the self-regulatory principles would be punished by ‘delisting [from app stores], relabelling [the app store listing to identify loot box presence], and in some cases, severe fines’ [44]. However, Ukie cannot actually do any of these by itself because it has no enforcement powers *per se*. This is because the self-regulatory principles did not provide any enforcement mechanisms empowering either Ukie or any other body to punish and deter non-compliance. (The first author [52] and other academics invited by DCMS had previously advised DCMS and Ukie to establish a robust enforcement framework to avoid this problem, but that advice was not taken.) Ukie must therefore rely on either app stores (e.g. Apple and Google) or the relevant age rating organization (i.e. PEGI and the IARC) to enforce the rules based on the terms of private contracts agreed between video game companies and app stores or age rating organizations. Ukie itself, as a third party, has no power to enforce those contracts. However, the present results demonstrate that none of the sanctions threatened by Ukie have been implemented by those other stakeholders against widespread non-compliance by the most popular games operated by the most well-known companies. In fact, Ukie’s threat is rather empty upon closer inspection: nobody has the power to enforce against non-compliance with Principle 1 because that is not required by app store platform rules or age rating guidelines and is only required by the Ukie industry self-regulation. Further, Principle 4 is only partially enforceable by app stores and age rating organizations because although they do require the disclosure of loot box presence for newly released games, they provide for exceptions permitting older games to be non-compliant [20]. Finally, Principle 5 is only enforceable by app stores and not age rating organizations because the latter does not require the disclosure of loot box probabilities. The Ukie industry self-regulatory principles do not have a proper enforcement regime.

Second, the requirement to disclose loot box presence applies also as part of UK advertising regulations enforced by the ASA [49]. (In contrast, the ASA specifically decided *not* to impose and enforce the requirement to disclose loot box probabilities [34], despite being requested to do so by stakeholders and despite other European bodies that are responsible for interpreting and enforcing equivalent rules having decided to require probability disclosures [53–55].) Although the ASA has taken limited enforcement actions by ruling against companies in a few test cases [26,27,42,43,56,57] after being prompted to do so by previous academic research [20,49], this has not led to any significant improvements in compliance. This is partly because the ASA, being an industry self-regulator, has very limited powers when sanctioning companies for non-compliance: the usual punishment is for the company to be criticized in a ruling published online. Quite often, even that does not occur because the ASA has frequently decided to resolve obvious cases of non-compliance by providing informal advice to the company without publishing any materials that may be detrimental to the company’s reputation and public image and thereby act as a deterrence against non-compliance by both that company and other companies. Companies that have been repeatedly ‘advised’ by the ASA (e.g. Supercell and Electronic Arts) continue to contravene advertising rules, demonstrating the ineffectiveness of the ASA’s enforcement regime. In theory, the ASA could refer repeatedly non-compliant companies for more severe sanctions, including criminal prosecution, by consumer protection regulators [58,59]. However, that has not occurred as no video game company appears in the recorded list of companies that have been so referred [60]. Further, by informally resolving complaints, the ASA also makes it difficult for a public record of repeated non-compliance to be established, as no information is publicly provided at all. Technically, the ASA has a more formalized informal resolution regime that does at least publish the name of the offending company, the number of complaints and the date of the resolution, even though no other information is given. However, the ASA has chosen to informally resolve loot box-related complaints at a level that is further below that, publishing no information whatsoever.

Third, the UK consumer protection regulators (specifically, the CMA and Trading Standards) are empowered to enforce against the non-disclosure of loot box presence and of loot box probabilities because these constitute separate misleading omissions under consumer law. A company engaging in such unfair commercial practices commits an offence under the Consumer Protection from Unfair Trading Regulations 2008 (Regulations 6 and 10), which are to be replaced by substantially identical provisions of the Digital Markets, Competition and Consumers Act 2024 (Sections 227 and 237(2)). These two regulators are not known to have taken any enforcement actions against obvious and widespread non-compliance with consumer protection law by video game companies. Even if informal and not publicly known enforcement actions were taken, these have not been effective at substantially improving the consumer experience. The criminal prosecution of a well-known company could more effectively deter non-compliance.

**Figure 1.**
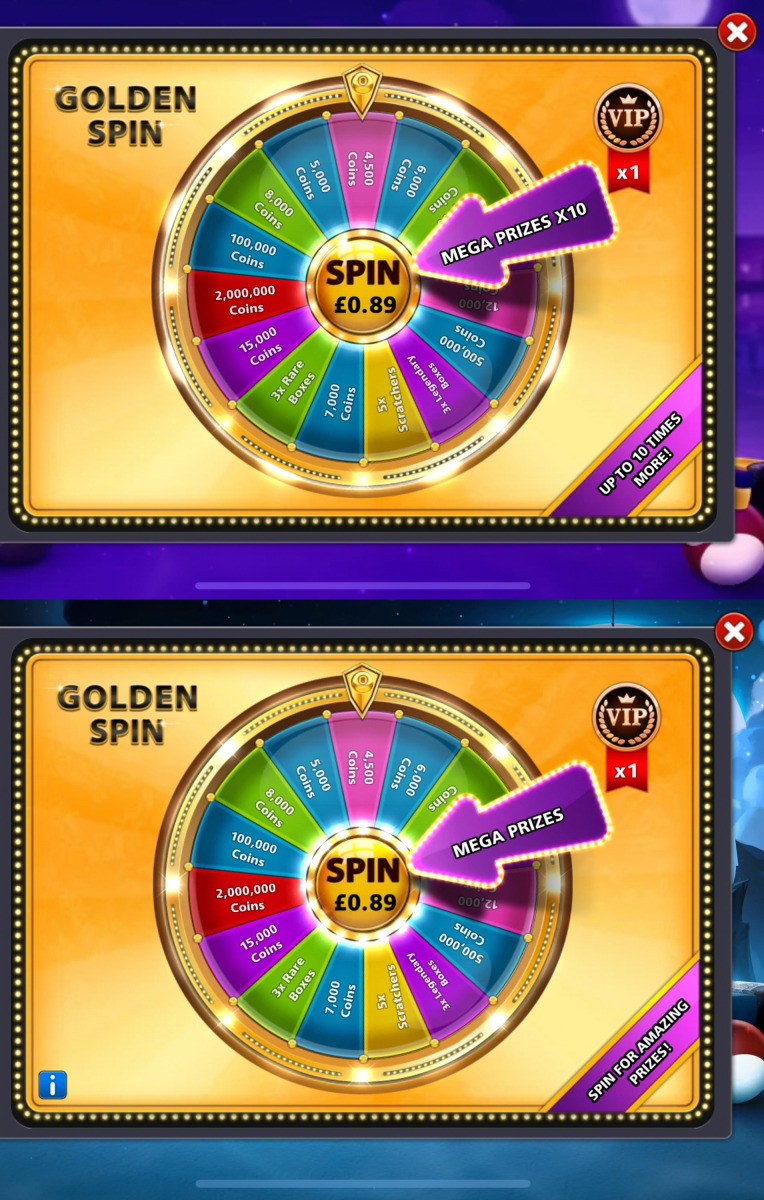
Probability disclosures were made more prominently in *8 Ball Pool* when it was assessed as part of the July sample after an advertising complaint was filed against the company following data collection for the January sample. © 2024 Miniclip.

The widespread non-compliance observed here should be addressed through better enforcement. As mentioned above, the ASA taking limited enforcement actions against a few companies has led to better compliance by those companies: for example, Hutch Games previously did not disclose loot box presence on the Apple App Store product listing page for *F1 Clash*, but it did so after a complaint was made against the company to the ASA by the first author [26]. Another complaint filed against Miniclip resulted in probabilities being disclosed more prominently in *8 Ball Pool* with the addition of a small blue [i] button that led directly to the probability disclosures when the January and July sample results are compared, as shown in figure 1 (the advertising language was also amended to remove misleading and unsubstantiated claims), even though no formal ruling has yet to be handed down after the company was informed of the concerns raised.

Similarly, complaints filed against companies based in Ireland to the Irish advertising regulator have led to, for example, Playrix subsequently disclosing loot box presence for *Township* [61]. A complaint was filed in The Netherlands against MY.GAMES to the Dutch advertising regulator in relation to *Rush Royale*, which has led to loot box presence and also probabilities being subsequently disclosed. These remedial actions were taken after the companies received notice of the complaints but before any formal rulings were published by the regulator. These examples illustrate how compliance could be improved when individual companies are appropriately pressured. Regulators need not necessarily enforce strict sanctions against non-compliance in every case (e.g. criminal prosecution): informal enforcement actions, such as informing companies about their obligations and threatening sanctions, may well be sufficient in many cases.

Notwithstanding, if informal enforcement fails to discernibly improve compliance and consumer protection (e.g. Supercell and the industry more broadly continuing to advertise misleadingly and illegally in the UK by failing to disclose loot box presence [49]), then regulators must stop relying on ineffective informal enforcement, formally enforce the rules (e.g. investigating and eventually publishing rulings against the company’s rule-breaking) and impose stricter sanctions. A combination of both may be the most cost-effective and efficient manner of enforcement: an informal admonishment for a first offence, but severe punishments against repeated offenders. In any case, regulators ought to do their duty and take enforcement actions of their own volition. It is unsustainable to rely on individual academic researchers volunteering their time and resources to pursue individual cases across different countries. Specifically, the ASA is called upon to take more strict actions against breaches of UK advertising rules by video game companies, including considering extending its regulatory ambit to ensure all ads targeting UK consumers are regulated (at present, non-paid for advertising, such as UK app store product listing pages, by non-UK-based companies are not regulable, but those by UK-based companies are, thus unequally treating different companies and meaning that the ASA cannot reliably force loot box presence disclosures to be displayed for many popular games).

As mentioned above, a number of leading video game companies were invited by the UK Government to be members of the working group that designed the industry self-regulations [16]. Naturally, these companies should be held to a higher standard: one ought to follow one’s own rules before expecting others to do so. However, non-compliance by working group members was detected. Examples include but are certainly not limited to the following. Hutch Games did not implement technical measures to prevent loot box purchasing by under-18s without parental consent, contrary to Principle 1. Activision Blizzard King failed to disclose loot box presence on *Call of Duty: Mobile*’s Apple App Store listing page, thus violating Principle 4. In breach of Principle 5, Electronic Arts failed to disclose probabilities for certain loot boxes in *Golf Clash*, and Take-Two Interactive also failed to do so in *WWE SuperCard*. Foreign companies that are unfamiliar with the rules may be somewhat excused for failing to comply, but even companies with a substantial presence in the UK that were invited by the UK Government to design the Ukie industry self-regulatory principles and therefore certainly had full knowledge of them failed to properly comply with those rules. These working group members’ non-compliance is more culpable.

It is expected that some members of the video game industry may disagree with the current study’s reasoned decision to exclude Apple’s Ask to Buy feature as satisfying the requirement of Principle 1. The rationale has already been stated. In addition, evidence was found that at least one video game company specifically instructed players and parents to turn off the feature because it did not work, which by implication would also disable the feature for other games if turned off system-wide. The Apple App Store product listing for *DRAGON BALL Z DOKKAN BATTLE* by Bandai Namco stated:

[Note About ‘Family Sharing’]

This application currently ***does not support the ‘Ask to Buy’ feature included with Family Sharing***. As a result, using this feature to make an in-app purchase on a device with Family Sharing enabled may result in an error. ***We ask that you do not use the ‘Ask to Buy’ feature when purchasing items until we have updated our application to support this feature***. [Emphasis added]

This further calls into question the reliability and adoption rate of Apple’s Ask to Buy feature: one company is literally instructing players and parents to *not* use the feature, contrary to and, in fact, in direct opposition to Principle 2 of the Ukie self-regulatory principles: ‘Drive awareness of and *uptake* of technological controls’ (emphasis added) [15]. Parents are being actively *discouraged* from using parental control features.

Another aspect of the present study that may be criticized is that an arguably overly broad definition for ‘loot boxes’ was used. More specifically, in many games, the main loot box mechanic would disclose probabilities, but other, perhaps more minor in-game purchases involving randomization that are less recognizable as a loot box would fail to disclose probabilities, thus contravening Principle 5. This is a phenomenon that has also been observed in Mainland China [48]. Some members of the industry may disagree as to whether those latter mechanics would constitute a loot box requiring disclosure. However, the present study used the exact definition set out in the Ukie self-regulations, which was broad and arguably imprecise:

In this document:

*‘**Loot Box**’* means a video game mechanic that provides random in-game virtual items to players in exchange for real-world money or in-game virtual currency. This document does not apply to a loot box that is purely earned through gameplay.

*‘**Paid Loot Box**’* means a Loot Box that is either purchased using real-world money or acquired using virtual currency that itself has been purchased. [Emphasis original] [15]

All loot boxes recognized as a loot box during the coding process satisfied the above Ukie definition. Nearly all loot boxes studied were clearly intended to repeatedly generate revenue from players. However, for an extreme outlier example, the mechanic that satisfied this definition in *Minecraft* was most certainly not a mechanic that these rules were intended to apply to. The player was able to buy with real-world money a game world in which random rewards could be obtained by defeating enemies. The player is able to recreate the game world an unlimited number of times and thus defeat the enemies and open the ‘loot box’ an infinite number of times (in contrast to nearly all other loot boxes that required purchase using real-world money to increase the finite number of openings and resultant random rewards). That *Minecraft* mechanic satisfied the definition just like a paid content update for a video game introducing new enemies whose defeat may randomly reward players with different results (e.g. the *Shadow of the Erdtree* update for *Elden Ring*). This was not intended but is the result of the poor drafting of the Ukie self-regulations. An improvement might be to amend and require the purchase to be repeatable or that opportunities to obtain random rewards are finite unless further payments are made.

Members of the video game industry who were not involved with the design of the Ukie loot box self-regulations are entitled to feel that Ukie has failed to advance their interests: the industry representative body published ill-defined rules that are difficult to comply with and made impossible promises to the UK Government and the UK public that could never have been kept, especially considering the lack of direct enforcement powers. Ukie and many members of the working group not only harmed consumers and harmed their own reputation through this exercise but also brought much bad press for the video game industry overall. Many members of the industry are not involved with or benefit from loot boxes whatsoever (but do pay to be represented by Ukie); many are even in favour of stricter regulation of loot boxes. In recent years, the industry has finally broken through some of the stigma associated with, for example, violent video games [62,63] and addiction [64,65], by demonstrating the potential benefits of video games not just to players [66] but also to the arts and the economy [67]. It is particularly disappointing that a trade body meant to represent the *whole* video game industry has compromised the *overall* interests of the video game industry so that the *minority* interests of a few leading video game companies (e.g. Electronic Arts) to continue profiting from loot boxes could be protected, at least for some more time.

**Figure 2.**
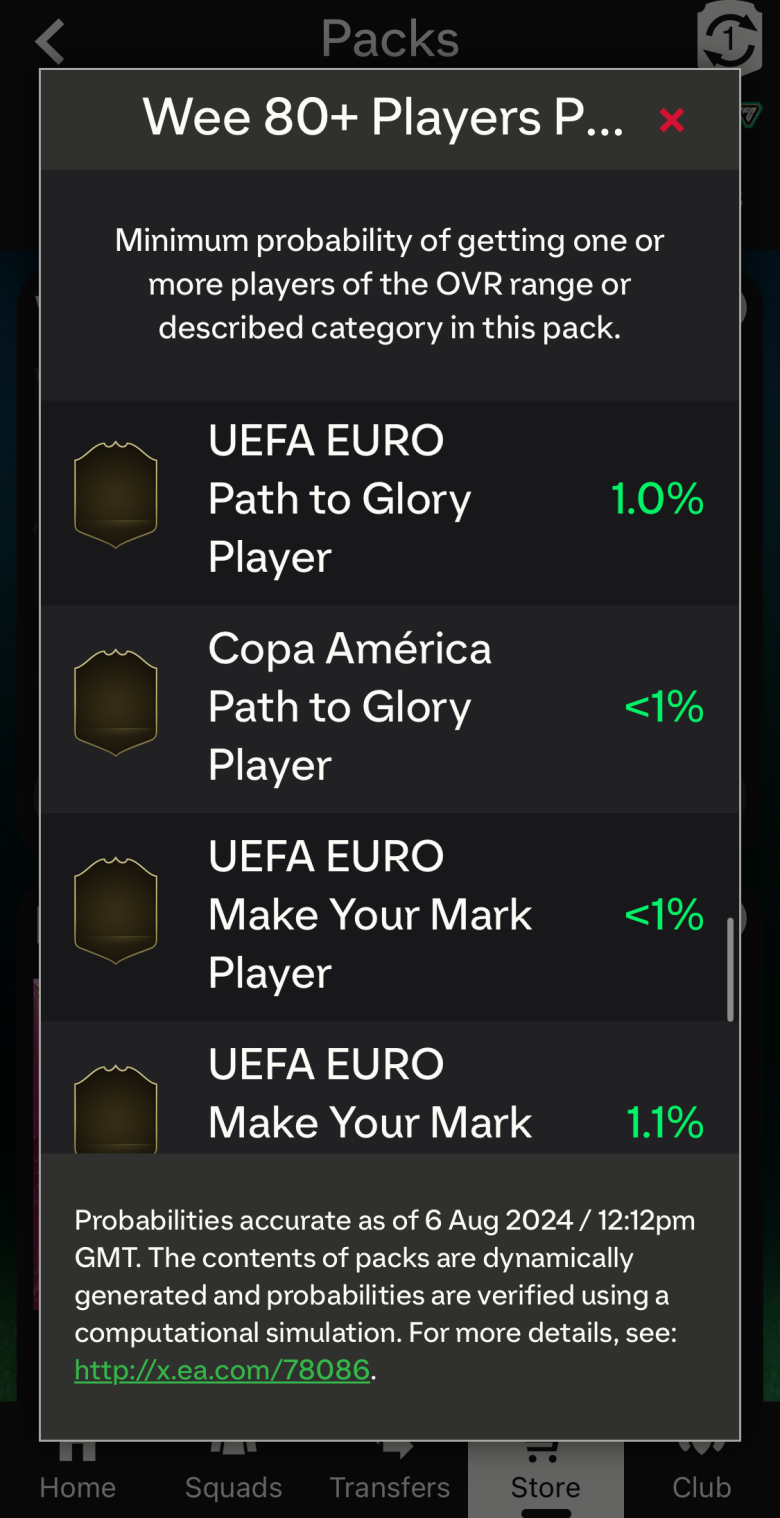
The probability for obtaining certain rewards was disclosed in insufficient detail as ‘<1**%**’ in *EA SPORTS FC 24 Companion*. **©** 2024 Electronic Arts.

Finally, in accordance with the Ukie self-regulation, probability disclosures were recognized as having been made even if, for example, the company merely informs the reader that the probability for getting a certain item is ‘<1%’, as occurred in *EA SPORTS FC 24 Companion* by Electronic Arts and shown in figure 2. Such an imprecise disclosure is unlikely to be recognized as legally compliant. In fact, Electronic Arts had to provide more detailed disclosures (0.045% instead of <1% [68]) when South Korea stopped relying on industry self-regulation and enforced its new law requiring loot box probability disclosures [69]. Therefore, the probability disclosure ‘compliance’ rate reported herein is reflective merely of compliance with the Ukie rules and not consumer law that should be enforced by the CMA and Trading Standards, which likely requires a higher standard and would deem more games as having been non-compliant.

**Figure 3.**
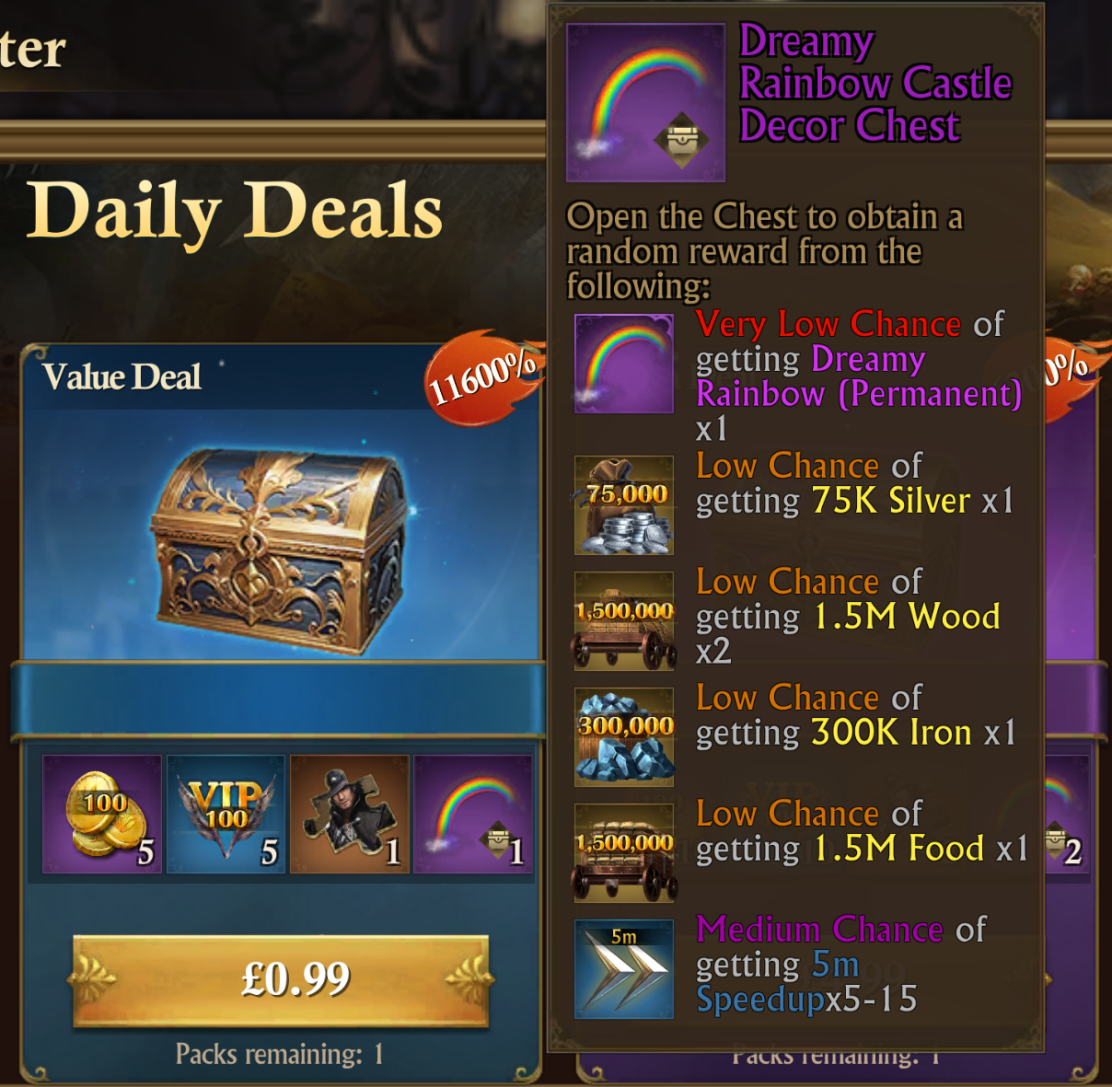
The purported probability disclosure for a loot box in *Guns of Glory* stated the probabilities using adjectives, such as ‘Very Low Chance’. © 2024 FunPlus.

For the avoidance of doubt, some purported probability disclosures were not deemed as compliant, as shown in figure 3. In this example, the probabilities for a loot box in *Guns of Glory* were disclosed as ‘Very Low Chance’, ‘Low Chance’ and ‘Medium Chance’, which were only relative to each other and not informative. The companies failed to provide effective and usable probability information as to the likelihood of obtaining different rewards. It is concerning that these companies are aware of their obligation to disclose loot box probabilities but chose to ‘comply’ maliciously to the detriment of consumers while also disrespecting the authorities of industry self-regulation and consumer law.

Important limitations were already set out where immediately relevant. Due to resource constraints, it was not possible to examine all games ‘immediately’ after the relevant dates upon which the highest-grossing lists were based, as it was preregistered (although a reasonable time gap was envisioned). This meant that, for example, some games were examined a mere 2 days after 18 January 2024; however, other games in the same sample were examined on 24 June 2024. That was a five month difference. The games that were examined earlier in January may have complied by June, so the results may have been different had they been examined later. It is known that at least one game (*June’s Journey: Hidden Objects* by the German company, Wooga) became compliant with the requirement to disclose loot box presence after its Apple App Store product listing page was first reviewed on 20 January 2024 but before 18 July 2024 (in fact, at some point before 25 April 2024, according to correspondence with the ASA, which had contacted the German advertising regulator at the first author’s request to enforce advertising regulations). The same applied to games that were examined immediately after 18 July 2024, as compared to those assessed in November 2024, because some companies may have taken some more time to comply, despite the one-year grace period that was already provided. Games that were examined earlier were therefore treated more unfairly in comparison to games that were assessed later, which had more opportunities to comply. However, all games were liable to be compliant at all relevant times irrespective of the date of the examination (except that games in the January sample were not required to comply with Principle 1 before it came into force), and all games in the January sample were examined within the relevant period (i.e. prior to 18 July 2024), so overall, the method was fair. A future study may consider recruiting a greater number of researchers to simultaneously examine different games within a tighter timeframe to further reduce bias.

## 5. Conclusion

The Ukie industry self-regulatory principles on loot boxes became effective in the UK from 18 July 2024 after a 1 year grace period following its publication on 18 July 2023 [15]. These were intended to better protect consumers, including young children, from potential harms (e.g. overspending money due to the product’s gambling-like nature). Regrettably, after 18 July 2024, no game with loot boxes (0.0%) sought to obtain explicit parental consent prior to enabling loot box purchasing by under-18s as required by Principle 1. Only 23.5% of games with loot boxes disclosed their presence on the games’ Apple App Store product listing page as required by Principle 4, and all of the games that did so disclosed in a manner that was difficult for consumers to access and arguably failed to meet advertising regulation and consumer law standards. A mere 8.6% of games with loot boxes consistently disclosed the probabilities of obtaining different rewards for all loot boxes found within 1 h of gameplay. The rules are not being actively enforced, and companies are permitted to break them with impunity: all of the games that were non-compliant remained non-compliant many months later, despite Ukie and the Apple App Store having been put on specific notice to demand corrections from the relevant companies or else delist those games.

Non-compliance is widespread. Enforcement is non-existent.

All relevant stakeholders are called upon to better enforce various rules intended to protect consumers, including young children, from potential harms associated with video game loot boxes. The Ukie industry self-regulatory principles are very poorly complied with and suffer from fundamental flaws, such as a lack of an effective enforcement framework. It has been reported that the then Conservative UK Government and the video game industry were ‘mired in circular arguments over the practicalities of administering [the self-regulation]’ with one insider describing the situation as an ‘absolute shit show’ [70]. The current and new Labour UK Government is advised against continued reliance on demonstrably ineffective industry self-regulation (both the Ukie loot box rules and the ASA advertising rules) to address the public’s loot box concerns.

Initially, it may have been sensible to try to quickly address concerns associated with novel technologies using industry self-regulation that does not require a protracted legislative process [71]. However, so-called ‘microtransactions’ or additional purchases associated with video games have been popularized for nearly two decades (since the horse armours were first sold to players of *The Elder Scrolls IV: Oblivion* in 2006 [72]), and more than 7 years have passed since the loot box issue first entered the Western public debate (i.e. the controversies surrounding the release of *Star Wars Battlefront II* in 2017 [73]), so the issues are no longer new. Since then, scientific knowledge about the potential harms of loot boxes has developed [4,74], and various industry self-regulations intended to appease policymakers and the public have also been repeatedly proven as poorly complied with and ineffective [19–21,49,51], including by the present study. Despite having been given ample opportunities to demonstrate corporate social responsibility, the video game industry has again and again failed to improve consumer protection or even comply with basic legal requirements (e.g. not hiding important information whose provision is required by consumer law). Video game industry self-regulation in relation to monetization is unreliable and must not be relied upon. South Korea recently replaced its previous loot box industry self-regulatory system with formal legal regulation, which has led to tangible benefits for the consumer (e.g. stricter rules and better enforcement leading to more detailed probability disclosures) [47,69,75]. It is high time real laws are passed, and pre-existing laws are enforced to properly regulate loot boxes and video games more broadly around the world.

## 6. Postscript

For the avoidance of doubt, this study has been conducted independently of the study being conducted by Public Group International Ltd (t/a PUBLIC) on commission from the UK Department for Culture, Media and Sport (DCMS) [76], for which the first author serves as an expert consultant. The results should be read in conjunction.

DCMS has not responded to the final results of this study. Ukie has decided to challenge the motivations for conducting this study and its results [77] without any basis and failed to admit and endeavour to address obvious failings, as the first author explained in his reply to Ukie [78]. Ukie has also decided to place heavy reliance on the forthcoming results of the PUBLIC study [77], which have not yet been published at the time of writing.

## Data Availability

The raw data and a full library of PDF printouts and screenshots showing, *inter alia*, the relevant Apple App Store webpage sections and in-game loot box purchase pages for each game are publicly available in the Open Science Framework [79,80]. Preregistrations are also available via the Open Science Framework [81,82].

## Authors' Notes

L.Y.X. plays and enjoys video games and broadly views the activity very positively, except for certain aspects (e.g. monetization) that he believes should be subject to more scrutiny. In terms of L.Y.X.’s personal engagement with loot boxes, he has played and continues to play video games containing loot boxes, such as *Hearthstone* (Blizzard Entertainment, 2014) until 2018 and *Genshin Impact* (miHoYo, 2020) and *Zenless Zone Zero* (miHoYo, 2024) since their initial release. He therefore engaged and continues to engage with non-paid loot boxes on a regular basis. However, he has never purchased any loot boxes with real-world money aside from negligible spending for research purposes to, for example, confirm the presence of paid loot boxes.
